# Beyond the Bony Fragment: A Review of Limbus Vertebra

**DOI:** 10.7759/cureus.60065

**Published:** 2024-05-10

**Authors:** Cosmin Nișcoveanu, Deria Refi, Bogdan Obada, Serban Dragosloveanu, Cristian Scheau, Radu Octavian Baz

**Affiliations:** 1 Department of Radiology, Sf. Apostol Andrei County Hospital, Constanta, ROU; 2 Department of Radiology and Medical Imaging, Faculty of Medicine, Ovidius University, Constanta, ROU; 3 Department of Orthopaedics and Traumatology, Sf. Apostol Andrei County Hospital, Constanta, ROU; 4 Department of Orthopaedics, Foisor Clinical Hospital of Orthopaedics, Traumatology and Osteoarticular TB, Bucharest, ROU; 5 Department of Orthopaedics and Traumatology, The Carol Davila University of Medicine and Pharmacy, Bucharest, ROU; 6 Department of Radiology and Medical Imaging, Foisor Clinical Hospital of Orthopaedics, Traumatology and Osteoarticular TB, Bucharest, ROU; 7 Department of Physiology, The Carol Davila University of Medicine and Pharmacy, Bucharest, ROU

**Keywords:** magnetic resonance imaging, ct (computed tomography) imaging, trauma & orthopedics, medical imaging diagnosis, bony fragment, spine, limbus vertebra

## Abstract

Vertebral limbus is a condition characterized by the intraspongious herniation of a portion of the nucleus pulposus. It is often asymptomatic, but it can sometimes cause nonspecific symptoms such as local pain and muscle spasm, or, in rare cases, radiculopathies, which is why it can be confused with vertebral fractures, spondyloarthropathies, infectious or tumoral processes. Early recognition of this pathology is preferable for a correct diagnosis and adequate treatment, the latter ranging from conservative approaches (such as personalized exercise programs and physical therapy) to surgical interventions reserved for severe cases with nerve compression.

## Introduction and background

Vertebral limbus is a condition characterized by the marginal intraosseous herniation of a portion of the nucleus pulposus, which is often asymptomatic, but it can cause nonspecific symptoms such as pain, localized muscle spasms, and radiculopathy. Vertebral limbus was first described by C.G. Schmorl in 1927, and its prevalence remains unknown [1]. The characteristic radiological appearance is represented by a detached bony fragment, triangular in shape, with sclerotic margins, usually at the level of the anterosuperior vertebral corner [1,2]. This review aims to revisit key aspects of vertebral limbus, from its suspected origins in immature spine trauma to its radiographic hallmarks, varied clinical manifestations, and treatment options.

## Review

Anatomy and physiopathology

Normally, the developing cartilaginous endplate (usually ossifying between six to nine years old), fuses completely with the main body of the vertebra by 18 to 20 years old. Vertebral limbus forms when a portion of the nucleus pulposus from an intervertebral disc pushes through a weak spot in the adjacent (frequently lower) vertebra. Thus, the prolapsed disc material prevents the physiological fusion of the endplate and the vertebral body, causing the endplate to develop as a separate, triangular bone fragment [3].

While the research on limbus vertebra's etiology is ongoing, literature studies have listed biomechanical factors such as the COL11A1 gene [4], genetic predisposition [5,6], developmental abnormalities [7], and sporting experience [2] as possible contributive aspects.

Clinical presentation and diagnosis

Vertebral limbus often presents a hidden challenge due to its diverse clinical picture. While asymptomatic cases are not uncommon, symptomatic individuals can experience a range of complaints, mainly low back pain, muscle spasms, and stiffness, further hindering mobility. In some cases [8-11], patients might experience radiculopathy, leading to pain, numbness, or tingling in specific leg regions due to nerve compression. The location of the limbus fragment plays a crucial role in symptom generation. Posterior vertebral limbus, due to its proximity to spinal nerve roots, is more likely to cause nerve compression and the associated radiating pain [11-13].

However, the non-specific nature of these symptoms often leads to confusion with other spinal conditions, highlighting the crucial role of accurate diagnosis to guide proper management.

When it comes to diagnosis, X-rays are the first line of investigation. Typically, X-ray reveals a characteristic triangular bone fragment with clear borders. This fragment sits adjacent to a corresponding defect in the vertebral body, but the fragment size often doesn't perfectly match the defect. Younger patients can present with more challenging aspects. In children and adolescents, the fragment might appear irregular and blurred, potentially mimicking infections or tumors [14]. Posterior vertebral limbus manifests as a bone defect in the posterior corner of the vertebral body along with a wedge-shaped osseous fragment behind or extending from the vertebral endplate. Usually, no additional tests are needed to make the diagnostic, except in the case of atypical images.

**Figure 1:**
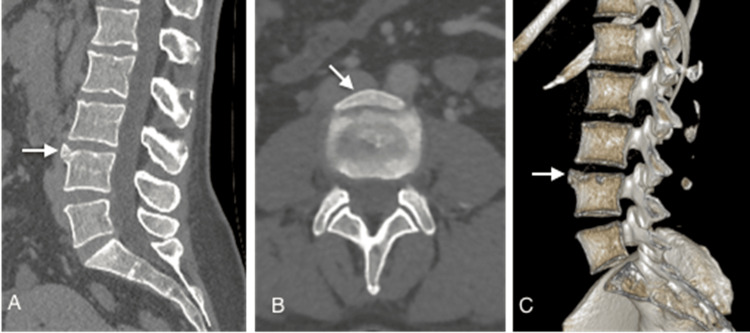
Sagittal (A), axial (B), and volume-rendered 3D reformatted images (C) show an L4 limbus vertebra with a bony triangular fragment (arrow) and sclerotic margins located adjacent to the anterosuperior corner of the L4 vertebral body. The image credits belong to the authors.

**Figure 2:**
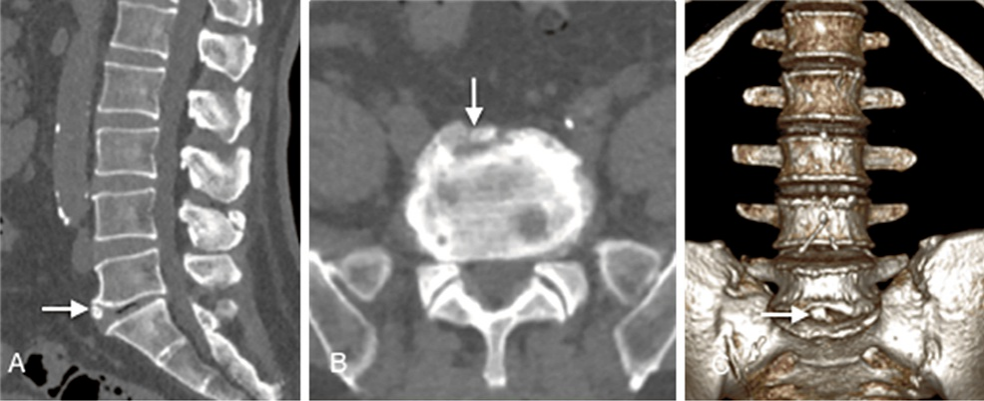
CT scan sagittal (A), axial (B), and volume-rendering (C) reconstructions of a patient with degenerative changes in the lumbar spine. At the level of the antero-inferior corner of the L5 vertebra, a triangular bone projection (arrow) is seen, almost completely separated from the vertebral body, pointing towards the likelihood of an osteophyte. Note other small anterosuperior osteophytes located at L4 and L5 levels. The image credits belong to the authors.

On computed tomography (CT) scans, vertebral limbus presents with characteristic findings that aid in diagnosis. The key feature is a well-defined, triangular, or round bone fragment adjacent to the anterosuperior corner of a vertebral body, most commonly in the mid-lumbar spine, but it can also be located near the inferior and posterior margins and at different vertebral levels [15]. This fragment exhibits sclerotic margins and appears distinct from the nearby vertebra, which shows an irregular-bordered defect related to the fragment's location (Figure 1). An anterior osteophyte that appears in a degenerative context is frequently included in the differential diagnosis, favoring factors being the absence of trauma and symptoms, as well as other findings of discarthrosis [16] (Figure 2). In cases of posterior limbus vertebra CT is the best option for depicting the bony defect in the posterior corner of the vertebral body and the retropulsed limbus fragment both on axial images and sagittal reformats [8].

**Figure 3:**
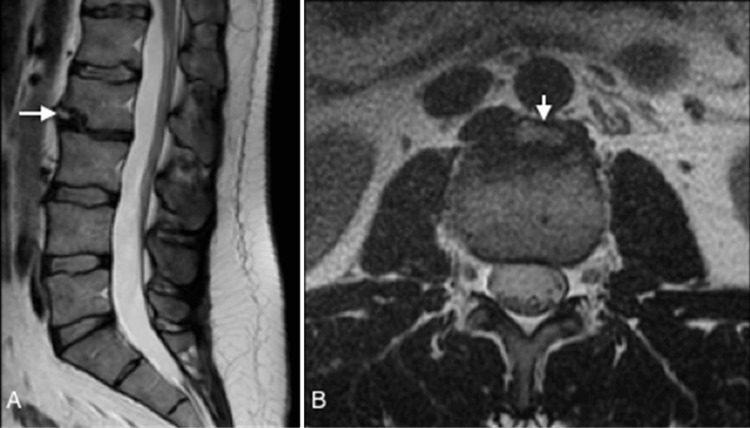
Sagittal (A) and axial T2-weighted (B) images show a detached fragment (arrow) at the level of the antero-inferior corner of the L2 vertebra, due to the intraosseous herniation of an adjacent portion of the nucleus pulposus The image credits belong to the authors.

**Figure 4:**
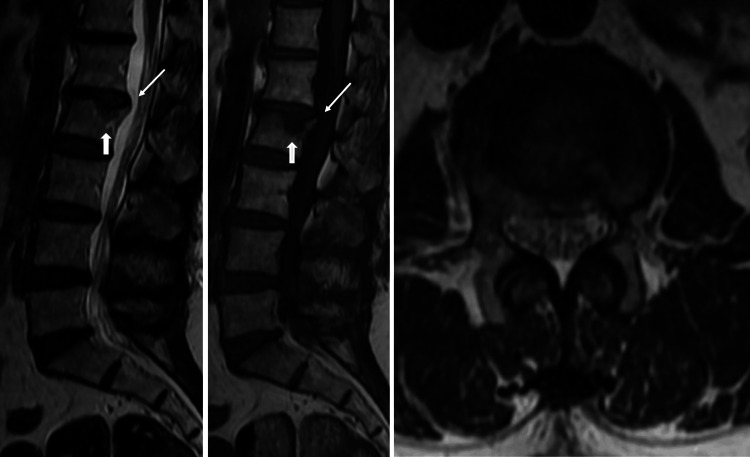
Sagittal T2 (A) and T1 (B), and axial T2-weighted (C) images show a subchondral geode (thick arrow) in the superior plateau of the L2 vertebra, which associates a small disc herniation and posterior limbus of the superior vertebral corner (thin arrow). The image credits belong to the authors.

Magnetic resonance imaging (MRI) plays an important role in detecting and characterizing vertebral limbus, offering superior visualization compared to X-rays. Key findings on MRI include a bony fragment developed adjacent to a corner of the vertebral plate, also revealing the absence of bone edema, ruling out fractures (Figure 3, Figure 4). A significant advantage of spine MRI is the superior evaluation of intraspinal lesions and the degree of associated spinal stenosis [17].

In many cases, patients with limbus vertebra have concurrent Schmorl nodes (cephalad or caudad intravertebral disc herniation). These can occur due to the close relationship between the pathogenesis of the two types of lesions. Schmorl nodes are best visualized on sagittal series and frequently have similar signal intensity to the nearby intervertebral disc, and a very thin peripheral rim of osteosclerosis [18]. In cases of acute herniation, the surrounding bone may exhibit marrow edema and peripheral enhancement. At times, the gas extrusion sign may be identified [13,18].

There are also some limitations in the identification of limbus vertebra through MRI. When the bone fragment is large enough, the central marrow fat hypersignal on T1- and T2-weighted images allows for correct diagnosis. However, when the lesion is small and only cortical bone is present, the low signal intensity limbus fragment is often difficult to evaluate and can be mistaken for osteophytes, disc calcifications, or focal ossifications of the anterior longitudinal ligament, thus leading to an inaccurate diagnosis. The careful distinction of the direction of disc herniation, whether intravertebral through the ring physis or intraspinal through the posterior annulus fibrosus, is the key to accurate diagnosis of posterior limbus vertebra [19].

Management strategies

While conservative therapy remains the mainstay for symptomatic vertebral limbus, the potential benefits of specific interventions like balneotherapy and kinesiotherapy warrant further exploration [20].

Tailored exercise programs incorporating core strengthening, flexibility, and postural correction can lead to significant pain reduction and improved functional capacity [21]. Similarly, analgesic and anti-inflammatory effects of warm water therapy, particularly when combined with physical therapy exercises show definitive potential [21,22].

However, in cases of posterior limbus vertebra associated with nerve root compression or when conservative treatment fails, surgical interventions may also be considered. The exact surgical approach depends on various factors, including the location and size of the fragment, the presence of instability, and associated pathologies. Any and all anatomical variants and morphological features of the spine, musculature, vessels, and adjacent structures should be adequately reported when surgery is considered, in order to prevent intraoperative accidents [23-25]. Preoperative planning using dedicated AI-enhanced software and intraoperative 3D visualization of patient imaging can increase the success rate of the surgical approach [26-29]. Using 3D-printed models may be of particular use when important spine deformities are associated [30-34]. Orthopedic surgeons should also consider that nerve injury can occur during treatment and is dependent on the surgical approach, patient risk factors, and initial severity of neurological deficit [35,36].

Discussions

The early development phases of vertebral limbus occur during childhood and adolescence, a period marked by the ossification of vertebral apophyses that eventually fuse with the vertebral body by adulthood [13]. Thus, vertebral limbus is a consequence of immature spine trauma. Vertebral apophyses ossify during childhood and adolescence until they eventually fuse with the vertebral body through skeletal maturation around the age of 18 [37]. During this vulnerable period, chronic stress, trauma, or congenital abnormalities can lead to an intraosseous marginal herniation of the nucleus pulposus between the annulus fibrosus and the adjacent vertebral body, resulting in anterior or posterior vertebral limbus. The separated fragment represents a segment of the annulus fibrosus that ossifies separately without fusing with the rest of the vertebra [38,39].

In most cases, it affects the anterosuperior corner of a single vertebra, usually at the level of the lumbar spine, but it can also be found in the inferior and posterior portions of any vertebral body. It is thought that vertebral limbus is frequently located at the anterosuperior edge of the vertebral body, secondary to the fact that the superior vertebrae are smaller than the adjacent inferior ones. During loading of the back in flexion, it is likely that the anterior portion of the intervertebral disc is forced into the superior plate of the underlying vertebra [40].

Vertebral limbus may not be an isolated entity. A review of the literature shows studies suggesting that it is not uncommon for vertebral limbus, Schmorl nodes (i.e. disc herniations within the vertebral body), and atypical Scheuermann's disease (i.e. growth plate irregularities in the spine) to coexist in the same patient [18,40-42]. In their study, Huang et al. found concurrent vertebral limbus and Schmorl’s nodes in 32% of the patients included in the study [8].

The pathophysiology of vertebral limbus is similar to that of Schmorl nodes and Scheuermann's disease, in which nuclear material herniates more centrally and at multiple lower thoracic levels [18,43]. This association relies on a similar underlying mechanism, i.e. a weakness in the vertebral endplate, the bony interface between the disc and the vertebral body. This vulnerability allows for disc material to protrude inwards in various ways. When the protrusion occurs centrally and at multiple lower thoracic levels, it manifests as Schmorl nodes. If the weakness is localized at the junction between the cartilaginous endplate and the bony rim, especially under increased axial loading, a vertebral limbus can develop. This shared mechanism explains why young individuals are more susceptible to these types of intravertebral disc herniations compared to the more common intraspinal disc herniations [42].

Most patients with vertebral limbus are asymptomatic and it is often an incidental finding on a lateral radiograph of the spine. However, a review of the literature revealed atypical variants that presented with acute or chronic low back pain, paraspinal muscle spasms, and radiculopathies [5,11], or mimicking fractures [44,45]. Posterior vertebral limbus is more likely to be symptomatic because it can cause spinal nerve root compression [3,8,9,46].

The search for the root cause of vertebral limbus has led researchers to explore various risk factors, such as genetic predisposition. For instance, the TT genotype of the COL11A1 gene is associated with decreased resistance of the growth plate [4]. Additional risk factors may be represented by patient engagement in high-performance sports starting at a young age where the risk of developing degenerative disc disease is higher [17].

Additionally, engagement in high-impact sports during childhood and adolescence could lead to an increased risk of developing degenerative disc disease, potentially favoring vertebral limbus formation [2,5-7].

The typical radiographic appearance of vertebral limbus in adults is a small, triangular, or round bone fragment with sclerotic borders. The adjacent vertebral body has a bone defect with sclerotic and irregular margins that does not completely correspond to the fragment described above. Further imaging studies are used when the radiographic diagnosis is uncertain, especially in cases of posterior vertebral limbus, in which the lower lumbar vertebrae overlap with other pelvic structures [40].

Plain X-ray films show significant limitations and may only correctly diagnose posterior limbus vertebra in up to 50% of cases. The main failures are recorded at the level of L5 and S1 vertebrae, due to the superimposition of the pelvic bones and relatively small lesion sizes that are difficult to visualize, therefore superior imaging methods might be required for a correct diagnosis [47].

MRI emerged as a valuable tool for diagnosing vertebral limbus by successfully describing its characteristic appearances [48]. Key advantages of MRI over X-rays are its ability to differentiate vertebral limbus from a vertebral body avulsion fracture and provide additional information about intervertebral disc pathology, the spinal canal, and the presence or absence of inflammation and bone edema [49].

The differential diagnosis of vertebral limbus includes vertebral fractures, focal calcifications of the anterior and posterior longitudinal ligaments, infectious processes, calcifications of the intervertebral discs, tumoral masses, Schmorl nodes, and vertebral osteophytes [45,50].

Vertebral Fractures

While both vertebral limbus and fractures might appear as bone fragments on X-rays, the presence of sclerotic margins in the case of vertebral limbus helps to differentiate it from an acute vertebral avulsion fracture.

Calcifications

Calcifications in ligaments or discs can mimic the appearance of vertebral limbus on X-rays. However, a careful evaluation by a radiologist and potentially an MRI scan can help differentiate between the two entities.

Infectious Processes

Infections of the spine are relatively uncommon, but they can sometimes present with features similar to vertebral limbus. The absence of abnormal contrast enhancement helps differentiate limbus vertebra from infectious processes or tumoral masses.

Tumors

Spinal tumors represent an important differential diagnosis, especially if they show calcifications, such as in the case of meningiomas. While X-rays may not be ideal in establishing a definitive diagnosis, MRI is highly effective in identifying the relevant imaging features, such as contrast enhancement, solid component, restricted diffusion, and other factors, therefore allowing for proper distinction from vertebral limbus.

Osteophytes

Osteophytes are bony outgrowths that can develop on the edges of the vertebrae. While they don't typically cause symptoms on their own, they can sometimes be confused with vertebral limbus on X-rays. However, their location (typically on the edges of the vertebral body) and the absence of a corresponding defect usually help in the differential diagnosis.

When vertebral limbus is an incidental finding and the patient is asymptomatic, no treatment is necessary. For symptomatic patients, treatment is generally limited to conservative measures. The therapy focuses on pain relief and management using medications like muscle relaxants, pain relievers, and non-steroidal anti-inflammatory drugs. Physical therapy (kinesitherapy) plays a crucial role in strengthening core muscles and improving spinal flexibility, ultimately reducing pain and preventing future issues [51-53]. Additionally, balneotherapy, a form of spa therapy that utilizes mineral-rich waters and therapeutic baths, might offer additional benefits in managing pain and promoting healing [22,54-56]. Investigating the potential synergistic effects of combining balneotherapy and kinesitherapy could prove valuable in managing pain and improving long-term outcomes. Some studies report successful conservative management for asymptomatic cases and those with mild symptoms [46,57,58]. The patients with large apophyseal fragments had a greater chance of poor results.

Surgical management is rarely indicated and should only be considered in cases that cause nerve compression with refractory radiculopathy that fails to respond to conservative measures. Several surgical options exist, each with its own advantages and disadvantages:

Laminectomy

Laminectomy refers to the removal of the vertebral lamina in order to access and eliminate the determining factor of nerve compression [8]. A combination with the removal of herniated disc material might be appropriate in order to achieve supplementary decompression [59]. The decision to remove the limbus fragment alongside the disc remains a topic of debate. Some studies report better outcomes when the fragment is removed along with the disc, as leaving a mobile fragment might impinge on nerves and cause persistent pain [57,59,60]. Several techniques have been described for limbus fragment removal. Scarfo et al. [59] utilized a microdrill to break down and remove the fragment, while Asazuma et al. [61] employed a specialized tool called a shoe-shaped double-ended impactor to safely remove the fragment while protecting surrounding nerves and the spinal sac. Extended laminectomy might be needed for wider exposure, as shown by Krishnan et al. [60], where partial laminectomy was not adequate and total laminectomy was required to properly remove the fragments. Arthrodesis might be considered for added stability after extensive laminectomy [47,61-64].

Removal of Mobile Fragment Only

A less invasive approach involves only removing the mobile fragment of the limbus that is compressing the nerve [3,39]. This can be performed through a smaller incision and offers faster recovery times. Akhaddar et al. [39] suggest leaving immobile fragments in place and focusing on removing only mobile fragments that might impinge on nerves. Some patients might experience persistent low back pain after surgery if the limbus fragment is not addressed.

Discectomy and Fusion

In cases where the vertebral limbus is accompanied by a disc herniation and spinal instability, a discectomy and fusion might be necessary to address both issues and provide long-term stability [65]. Reports are generally showing good to excellent outcomes following surgery for posterior limbus vertebra. Recovery time and residual symptoms can vary depending on the severity of the case and the presence of a limbus fragment does not necessarily lead to a worse prognosis [39,57,61,63]. If significant bone defects are present due to trauma or associated pathologies, vertebroplasty can be performed using adequate medical-grade compounds [66-68]. Robotic surgery can also be considered as it is emerging as a safe, reliable technique and is increasingly applied in all surgical areas [69-71].

Vertebroplasty and Kyphoplasty

This minimally invasive procedure involves injecting medical-grade bone cement into the vertebral body to stabilize it. It can be considered in cases with significant bone defects due to trauma or associated pathologies and holds the potential to reduce pain and improve spinal stability. Kyphoplasty involves the additional step of creating a cavity for cement injection. While smaller lytic benign tumors might benefit from treatment with tricalcium phosphate mixed with hydroxyapatite as bone substitute, this low cost approach comes with the downside of being fragile and taking a longer time to integrate [67]. Performing kyphoplasty was shown to decrease the amount of cement leakage and improve the correction of kyphosis [68].

Robotic Surgery

Recent studies show percutaneous endoscopic lumbar discectomy, minimally invasive spinal surgery-transforaminal lumbar interbody fusion and microendoscope-assisted decompression surgery with resection of bony fragment emerging as minimally invasive techniques for treating a separation of lumbar posterior ring apophysis [72,73]. As robotic surgery continues to evolve and gain wider acceptance in various surgical fields, it might emerge as a future option for treating vertebral limbus, especially for complex cases. The potential benefits include increased precision, improved visualization, and potentially faster recovery times. However, more research and experience are needed before robotic surgery becomes a mainstream approach for vertebral limbus.

## Conclusions

Although vertebral limbus was first described radiographically almost a century ago, the condition remains relatively unknown to the medical community and definitive diagnosis can be challenging. It is often an incidental radiographic finding and can be mistaken for a variety of other pathologies. Recognition of vertebral limbus can help to limit unnecessary imaging studies or even avoid invasive procedures. While often masquerading as other spinal pathologies due to its diverse clinical presentation, recognizing its unique characteristics is crucial for accurate diagnosis and optimal management. The evolving landscape of conservative management strategies holds promise for offering effective, non-invasive options for those seeking relief from the more serious symptoms associated with this condition.
